# A Survey of Inhalant Use Disorders among Delinquent Youth: Prevalence, Clinical Features, and Latent Structure of DSM-IV Diagnostic Criteria

**DOI:** 10.1186/1471-244X-9-8

**Published:** 2009-03-08

**Authors:** Matthew O Howard, Brian E Perron

**Affiliations:** 1 University of North Carolina at Chapel Hill, Tate-Turner-Kuralt Building, Campus Box 3550, 325 Pittsboro Street, Chapel Hill, NC 27599-3550, USA; 2 University of Michigan, School of Social Work, 1080 S. University Avenue, Ann Arbor, MI 48109, USA

## Abstract

**Background:**

Inhalant use is among the most pernicious and poorly understood forms of adolescent substance use. Many youth in the juvenile justice system have used inhalants, but little is known about inhalant use disorders (IUDs) in antisocial youth populations. The purpose of this study was to examine the prevalence, clinical features, and latent structure of DSM-IV IUDs in a state population of antisocial youth.

**Methods:**

Cross-sectional survey conducted in 2003. Of 740 youth residing in Missouri State Division of Youth Services' (MDYS) residential treatment facilities at the time the study was conducted, 723 (97.7%) completed interviews. Eighty-seven percent were male, with a mean age of 15.5 (SD = 1.2). Nearly 4 in 10 youth (38.5%; n = 279) reported lifetime inhalant use. Youth ranged from very mildly to severely antisocial.

**Results:**

Of 279 inhalant users, 52 (18.6%) met DSM-IV inhalant abuse criteria and 79 (28.3%) met inhalant dependence criteria. Five of 10 IUD criteria were met by > 10% of the total sample. Latent class analyses demonstrated a substantial concordance between DSM-IV-defined IUDs and an empirically-derived classification based on responses to DSM-IV IUD diagnostic criteria.

**Conclusion:**

IUDs and constituent criteria were prevalent among youth in the juvenile justice system. Two groups of problem inhalant users were identified, symptomatic users-DSM-IV inhalant abuse and highly symptomatic users-DSM-IV inhalant dependence, which differed primarily in severity of inhalant-related problems. Inhalant screening, prevention and treatment efforts in juvenile justice settings are rarely delivered, but critically needed.

## Background

Inhalant use and formal DSM-IV inhalant use disorders (IUDs) remain a curious case study in the annals of psychiatric epidemiology. For more than thirty years, nationally representative surveys have identified inhalant use as among the most prevalent forms of adolescent substance use [1,2]. For example, findings from the 2006 Monitoring the Future survey indicated that 16.1% of U.S. 8^th ^graders had used inhalants, compared to 15.7% who had used marijuana/hashish, and 7.3%, 3.4%, 3.4%, and 1.4% who had used amphetamines, hallucinogens, cocaine, and heroin, respectively [3]. Wu and colleagues recently estimated that two million 12-to-17 year-olds in the U.S. had used inhalants based on data from the National Survey on Drug Use and Health [4].

A small corpus of animal studies, case reports, and clinical investigations further suggests that malignant health and social outcomes may attend adolescent inhalant use. Inhalant use has been implicated in documented cases of cardiac, renal, and liver toxicity and hepatorenal failure [5-7], bowel and bladder dysfunction [8], bone marrow suppression and reduced T-cell responsivity [9,10], irreversible congestive heart failure [11], severe neurological damage and cognitive dysfunction [12,13], effects in offspring similar to those of fetal alcohol syndrome [14], and a host of psychiatric, social, academic, and interpersonal functional impairments [15-19].

Despite these troubling findings, inhalant use remains poorly understood and the least studied of the psychoactive substances of abuse. In his presidential address to the College on Problems of Drug Dependence, Balster referred to inhalant use as "the hidden epidemic," noting the curious disjunction between the seriousness of the problem and the insubstantial scientific and clinical responses to it [20]. IUDs have been included in the *Diagnostic and Statistical Manual of Mental Disorders *(DSM) since 1980, although the brief discussion of these disorders in DSM-IV is testament more to the sheer absence of available findings vis-à-vis prevalence, course, subtypes, comorbid mental health and medical conditions, and specific clinical and sociodemographic features than a useful guide to the diagnosis of these disorders [21]. Additional research on IUDs is needed to support a more refined, substantive, and clinically useful discussion of IUDs in DSM-V.

One particularly important unresolved issue concerns the validity of the DSM-IV distinction between inhalant abuse and inhalant dependence disorders. Although the generic DSM-IV substance dependence disorder criteria set applied to most substance use disorders includes a criterion reflecting presence versus absence of a characteristic withdrawal syndrome, this criterion is not included in the inhalant dependence disorder criteria set. Further, as recognized in DSM-IV [21], other generic substance use disorder criteria may have been included in the IUD criteria sets inappropriately. For example, one generic substance dependence criterion assesses whether "a great deal of time is spent in activities necessary to obtain the substance, use the substance, or recover from its effects." However, it is unlikely that inhalant users spend a great deal of time accessing inhalants, given their ubiquity in the physical environment. We are not aware of any prior studies of the latent structure of DSM-IV IUDs. Findings of this nature could shed light on current proposals to move from a categorical (abuse vs. dependence) to unidimensional conceptualization of substance use disorder (i.e., dependence) in DSM-V [22-25]. Further research on IUDs in general is needed to improve screening, treatment and prevention.

The current study attempts to address these gaps in knowledge by focusing on a large sample of delinquent youth. Delinquent youth have among the highest rates of inhalant use identified in prior studies of community, clinical, and criminological samples. These findings suggest that delinquent youth may also be at comparatively high risk for IUDs. For example, Howard and Jenson identified lifetime inhalant use in 34.3% of 475 adolescents on probation in Utah [26]. Similar results were reported by Howard, Balster, Cottler, Wu, and Vaughn [27], who found that nearly 40 percent of 723 antisocial youth residing in Missouri State Division of Youth Services' residential rehabilitation facilities had used inhalants for the expressed purpose of becoming intoxicated. Howard et al. [27] examined characteristics of inhalant use in a state population of delinquent youth, including prevalence and patterns of use and differences between inhalant users and nonusers. Findings indicated that race/ethnicity, geographic area of residence, fearlessness, sucidality, and polydrug use distinguished inhalant users and nonusers in multiple logistic regression analyses.

Using the same data source as Howard et al. [27], Perron and Howard [28] examined correlates of IUDs. They found that delinquents with IUDs (particularly dependence) reported more extensive histories of antisocial behavior, trauma, substance-related problems, suicidal ideation, and higher current levels of anxious/depressive symptoms than did inhalant nonusers or users without IUDs. Youth with IUDs had significantly more substance-related problems and greater suicidal ideation than inhalant nonusers.

The current study extends Howard et al.'s [27] and Perron and Howard's [28] investigations by examining the prevalence and diagnostic performance of DSM-IV inhalant abuse and dependence disorders and their constituent criteria. These issues were not examined in the prior reports [27,28] but are important. More specifically, studies of community and clinical samples reveal that high rates of lifetime inhalant use do not necessarily imply high rates of DSM-IV IUDs [29,30]. However, statewide surveys [26,27] indicate that delinquent youth, particularly those in the juvenile justice system, may be at high risk for IUDs. Our review of the scientific literature pertaining to inhalants did not locate other prior investigations of DSM-IV IUDs in juvenile justice service populations. Thus, this investigation examined 1) the prevalence of DSM-IV inhalant abuse and inhalant dependence and constituent criteria in a state population of delinquent youth at substantial risk for these disorders, and 2) the latent structure of DSM-IV IUDs and concordance between DSM-IV IUDs and empirically-derived classes of adolescent inhalant users identified via latent class analysis of DSM-IV IUD diagnostic criteria. Results of this study may provide a foundation for improved screening, treatment, and prevention of IUDs in juvenile justice settings.

## Methods

### Survey Description

Findings of the present study are based on a survey conducted in 2003 of the population of current residents of the Missouri Division of Youth Services (MDYS), a statewide rehabilitation system comprised of 32 residential facilities. MDYS is the legal guardian of youth committed to its care by the state's 45 juvenile courts. Of the 740 MDYS residents at the time the survey was conducted, 10 were on furlough as interviewing commenced and 2 were transferred to another MDYS facility while interviewers were at a facility, but before they could be interviewed. Of the 728 adolescents available to interview, all agreed to participate. However, 5 interviews were discontinued; 4 adolescents displayed signs or reported symptoms of psychosis and one adolescent chose not to continue. The 723 adolescents who completed the interview constituted 97.7% of MDYS residents at the time interviewing was conducted, 99.3% of residents available for interviewing, and approximately 55.0% of adolescents committed to MDYS care in the prior year. Thus, the present study is virtually a census of the population of MDYS residents at the time the survey was undertaken and a large, representative sample of MDYS annual residents.

The MDYS service population is representative of delinquent youth in state-mandated residential care nationally with regard to the age, gender, and number of state youth incarcerated per 100,000 adolescents [31]. In 2000, 15.3% of MDYS residents were committed for status offenses (e.g., running away from home, truancy), 32.4% were committed for misdemeanors, 40.2% were committed for less serious (class C/D) felonies, and 12.1% were committed for the most serious (Class A/B) felonies [32].

Participation in the study was voluntary. Face-to-face interviews of adolescents were conducted using a comprehensive assessment inventory. All interviewers completed an intensive one-day training session and an interview editor was on-site at each facility as adolescents were interviewed to inspect interview schedules and thereby minimize interviewer omissions and errors. Interviews were conducted in capacious rooms that provided private areas where individual confidential interviews could be conducted simultaneously with between three and six adolescents. The sample recruitment protocol ensured that no adolescent who had completed the interview at one MDYS facility subsequently completed the interview at another MDYS facility.

Prior to participating in the study, each youth was asked to read an informed assent agreement, raise any questions they had about the study, and then sign the assent form if they felt their questions had been answered satisfactorily and had decided to participate. The informed assent form provided youth with detailed information about the study, assured them they were not required to participate, could end participation in the interview at any time, and that their legal status would not be affected by their decision to participate or not participate in the study. The informed assent form also provided potential participants with the name and telephone number of a non-study and non-university-affiliated advocate whom they could call for more information about the study. MDYS agreed that youth could use the telephone during business hours to call the advocate should they desire to do so and youth were informed of this agreement. The informed assent form was based on a template developed by the Washington University IRB and was specifically designed for use with pediatric populations.

As legal guardian of the youth, MDYS provided formal permission for youth to participate in the study. This study was approved by the MDYS Institutional Review Board, Washington University Human Studies Committee Institutional Review Board (with prisoner representative and otherwise operating in accordance with governing regulations for research on prisoners and youth), federal Office of Human Research Protection (as required for research on prisoners), and was granted a Certificate of Confidentiality by the National Institute on Drug Abuse (NIDA). All youth received an age-appropriate explanation of their privacy rights and a copy of the Washington University brochure, "Your Privacy Matters," as well as a copy of their signed informed assent agreement. Participants received $10.00, which was paid to their facility monetary accounts and a receipt for such payment along with 2 candy bars to eat during the interview. Youth were eager to participate in the study and highly valued the opportunity to temporarily escape their regimented daily routines and discuss their attitudes and life histories with interviewers. It is also true that word spread rapidly through each facility about the $10.00 and candy bars provided to recompense youth for their time and effort.

### Measures

All study participants completed the *Volatile Solvent Screening Inventory *(VSSI), a structured face-to-face interview. The VSSI requires approximately 45-minutes to complete and assesses demographic characteristics, medical history, lifetime and past year use of 55 volatile solvent inhalants (e.g., gasoline, paint thinner, etc.), other drug use and substance-related problems, current psychiatric symptoms, suicidality, trauma history, antisocial traits and criminal activity. A detailed description of the VSSI, its psychometrics, and a copy of the instrument itself is available in Howard et al [27]. Consistent with DSM-IV diagnostic guidelines, nitrite vasodilator or nitrous oxide use was not considered inhalant use for the purposes of this investigation [21].

Youth who reported lifetime use of one or more of the volatile solvents included in the VSSI, then completed the Comprehensive Solvent Assessment Interview (CSAI), also described and available in Howard et al [27]. The CSAI includes an assessment of the settings, modalities, peer and family networks, acute medical and social consequences, and other characteristics of adolescent inhalant use. Items and diagnostic algorithms from the *Diagnostic Interview Schedule *(DIS, v. IV) were included in the CSAI to assess for the presence of DSM-IV inhalant abuse and inhalant dependence disorders. The DIS evidences good-to-excellent reliability for assessment of substance use disorders [33]. IUD diagnoses were assigned in accordance with DSM-IV guidelines.

Adolescent inhalant users were assigned a lifetime inhalant abuse diagnosis if they met one or more of four DSM-IV criteria for inhalant abuse at some point in their lifetime (occurring within a 12-month period) and had never met criteria for DSM-IV inhalant dependence. Inhalant users were assigned a past-year diagnosis of inhalant abuse if they had met one or more inhalant abuse criteria in the year prior to entering MDYS residential care and had never met criteria for DSM-IV inhalant dependence. Adolescent inhalant users were assigned a lifetime inhalant dependence diagnosis if they met three or more of six DSM-IV inhalant dependence criteria within a 12-month period at some point in their lifetimes. A seventh generic DSM-IV substance dependence criterion – withdrawal symptoms – was not included in the inhalant dependence criteria set per specific DSM-IV guidelines (cf., p. 258 in DSM-IV [21]). A past-year inhalant dependence diagnosis was assigned to inhalant users who evidenced 3 or more inhalant dependence symptoms within the 12-month period immediately prior to their current MDYS episode of care.

### Analytic procedures

Univariate statistics were used to describe the prevalence of DSM-IV inhalant abuse and dependence diagnoses and constituent criteria. Empirically-derived classes of adolescent inhalant users were identified using latent class analysis based on youths' responses to DSM-IV IUD criteria. The first LCA analysis was carried out in an exploratory fashion to determine whether DSM-IV IUD criteria defined discrete latent classes of adolescent inhalant users. Specifically, rather than testing a previously specified, *a priori *class solution, a series of different models was evaluated with regard to indices of model fit and heuristic utility. A single-class model was examined first, and additional classes were specified until no further improvements in model fit were observed. The empirical fit of latent class models was assessed with the Bayesian Information Criterion (BIC), which measures goodness-of-fit, and entropy statistic, a measure of relative class purity. Finally, each latent class model was assessed by examining overall model results and diagnostics and by comparing youth in LCA-derived latent classes graphically in terms of their responses to DSM-IV IUD diagnostic criteria.

A second LCA analysis was confined to adolescent inhalant users who met DSM-IV criteria for lifetime inhalant abuse or inhalant dependence. The solution to this LCA was fixed to two classes. The concordance of empirically-derived class assignments of youth based on LCA of responses to DSM-IV IUD diagnostic criteria and assignments of youth based on standard application of DSM-IV diagnostic criteria was then assessed.

## Results

### Sample description

Eighty-seven percent of youth were male and approximately forty percent reported that their family received public assistance. Youth ranged from 11 to 20 in age (Mean = 15.5, SD = 1.2). Most study participants lived in small towns (39.5%, n = 286) or cities (39.1%, n = 283), with smaller percentages residing in suburban (13.8%, n = 100) or rural areas (7.5%, n = 54). Youth were racially diverse, with 33.0% (n = 238) reporting an African American identification, 7.7% (n = 56) a bi/multi-racial identification, 3.9% (n = 28) a Latino/Latina identification, and 55.4% (n = 400) a Caucasian racial identification. These descriptive findings and the inhalant use prevalence rate reported below were included in Howard et al [27]. Howard et al. [27] includes additional and more specific sample characteristics by age, grade, and proportion of respondents with a history of head injury, perinatal injury or illness, and physician-diagnosed mental illness.

### Inhalant use

More than one-third (38.6%; n = 279) of the total sample reported lifetime use of inhalants. The number of inhalants used ranged from 1 to 16 (Mean = 4.2, Median = 4.0, SD = 3.0).

### Prevalence of DSM-IV IUDs and Constituent Criteria

#### Inhalant abuse

Of youth with a lifetime history of inhalant use, 18.6% (n = 52) met DSM-IV criteria for lifetime inhalant abuse and 3.6% (n = 10) met diagnostic criteria for both lifetime and past-year inhalant abuse. Thus, more than four-in-five (i.e., 80.8%) youth who met lifetime inhalant abuse criteria did not meet criteria for past-year inhalant abuse.

Additional file 1 presents findings describing the prevalence of each DSM-IV inhalant abuse criterion in the overall sample, subsample of lifetime inhalant users, and subsamples of youth meeting DSM-IV Inhalant Abuse and Inhalant Dependence diagnoses, respectively. The most prevalent inhalant abuse criterion was recurrent inhalant use in situations in which it is physically hazardous, which was reported by approximately one-third of adolescent inhalant users. The least prevalent criterion was recurrent inhalant-related legal problems, reported by one-in-twenty inhalant users. Although the DSM-IV nosology excludes youth meeting inhalant dependence criteria from eligibility for an inhalant abuse diagnosis, a substantial majority (n = 63, 79.7%) of youth meeting inhalant dependence diagnoses would also have received an inhalant abuse diagnosis if dual IUD diagnoses were permitted by DSM-IV.

Our data do not allow us to examine the temporal ordering of inhalant abuse and dependence diagnoses. However, it is interesting to note that 115 of 279 (41.2%) inhalant users in this sample met lifetime DSM-IV inhalant abuse criteria if delinquents also meeting DSM-IV dependence criteria are included in this total. Of this number, 42 were free of past-year inhalant abuse, had never met dependence criteria, and thus appeared to have matured out of inhalant abuse; 10 youth met lifetime and past-year inhalant abuse criteria but not dependence criteria and may have experienced relatively recent onset of abuse or abuse that did not escalate to dependence; and 63 youth met criteria for both lifetime inhalant abuse and dependence.

#### Inhalant dependence

Among youth who reported inhalant use, 28.3% (n = 79) met DSM-IV criteria for lifetime inhalant dependence. Most youth meeting lifetime inhalant dependence criteria also met criteria for past-year inhalant dependence (87.3%; n = 69). The most prevalent inhalant dependence criteria were tolerance to inhalants, loss of control over inhalant use, and continued inhalant use despite knowledge of having a persistent or recurrent physical or psychological problem likely to have been caused or exacerbated by inhalant use. Even the least prevalent of the inhalant dependence criteria were met by more than one-in-five inhalant users.

The final column in Additional file 2 represents inhalant users classified with a Lifetime Inhalant Use Disorder Not Otherwise Specified (NOS). The diagnostic category was defined as inhalant users who met 1 or 2 lifetime inhalant dependence criteria but who did not meet diagnostic criteria for either inhalant abuse or dependence. These "diagnostic orphans" evidenced some symptoms of inhalant dependence but were not diagnosed with formal IUDs in the DSM-IV diagnostic system. Diagnostic orphans were not uncommon, comprising slightly less than one-fifth of the sample of lifetime inhalant users. Levels of inhalant dependence symptoms in this group were not insubstantial.

### Latent Structure of DSM-IV Inhalant Abuse and Dependence

**Figure 1:**
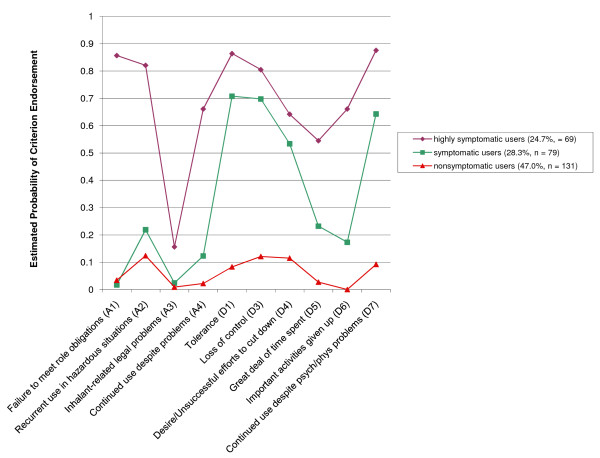
**Exploratory Latent Class Analysis of Lifetime Adolescent Inhalant Users' (N = 279) Responses to DSM-IV Inhalant Use Disorder (i.e., Abuse and Dependence) Diagnostic Criteria**.

The latent structure of DSM-IV inhalant abuse and dependence disorders was explored by first examining profiles of lifetime inhalant users based on DSM-IV-defined inhalant abuse and dependence criteria. The analysis was carried out using LCA. A three-class solution exhibited the best fit to the data (BIC = 2679, see Additional file 3) and was the most parsimonious and interpretable model with class purity (entropy = .83) similar to the 2- and 4-class models. As shown in Figure 1, each class of inhalant users exhibited an ordinally-differentiated profile with respect to their estimated probability of endorsing each IUD diagnostic criterion.

Of lifetime inhalant users, the estimated percentage of adolescents belonging to the largest group, labeled "nonsymptomatic inhalant users," was 47.0%. The percentage of inhalant users in this class was similar to the percentage of youth who reported lifetime inhalant use but who did not meet DSM-IV diagnostic criteria for inhalant abuse or dependence (i.e., 53.1%). This class exhibited the lowest probability of endorsing any of the IUD diagnostic criteria. Among nonsymptomatic inhalant users, the diagnostic criteria with the highest estimated probability of endorsement were inhalant use in physically hazardous situations (.12) and loss of control (.12) over inhalants.

An estimated 28.3% of adolescent inhalant users were assigned to a second group, labeled "symptomatic inhalant users." The percentage of inhalant users in this class was somewhat higher than the percentage of users classified with DSM-IV-defined inhalant abuse (i.e., 18.6%). Members of this class were more likely to endorse most inhalant abuse criteria compared to nonsymptomatic inhalant users, but were significantly less likely to endorse inhalant abuse criteria than members of the third LCA-derived group of inhalant users. Compared to nonsymptomatic inhalant users, symptomatic users also had substantially higher estimated probabilities of endorsing all six inhalant dependence criteria.

The estimated percentage of adolescents belonging to the third LCA-derived class of inhalant users, labeled "highly symptomatic users," was 24.7%. The proportion of inhalant users in this class was similar to the proportion of users classified with DSM-IV-defined inhalant dependence (i.e., 28.3%). This group exhibited significantly higher estimated probabilities of endorsing each DSM-IV IUD criterion than did the other two inhalant user classes. For the abuse criteria, the highest estimated probability of endorsement was for recurrent inhalant use resulting in a failure to fulfill major role obligations at work, school, or home (.86), followed by recurrent inhalant use in physically hazardous situations (.82). The probability of experiencing any of the DSM-IV inhalant dependence criteria was significantly elevated in this class compared to the classes of nonsymptomatic and symptomatic inhalant users. Members of this class evidenced a .55 to .88 probability of endorsing each DSM-IV inhalant dependence criterion.

Overall, 8 of 10 DSM-IV IUD criteria clearly differentiated nonsymptomatic inhalant users from symptomatic inhalant users and 6 of these were inhalant dependence criteria. All DSM-IV IUD criteria usefully differentiated symptomatic inhalant users from highly symptomatic inhalant users, especially recurrent inhalant use resulting in a failure to fulfill major role obligations, inhalant use in physically hazardous situations, continued inhalant use despite persistent/recurrent inhalant-related social/interpersonal problems, great deal of time spent in activities necessary to obtain, use, or recover from the effects of inhalants, and important social, occupational, or recreational activities given up because of inhalant use.

The correspondence of DSM-IV IUD diagnoses and LCA-based diagnoses of nonsymptomatic, symptomatic, and highly symptomatic inhalant users was examined in Additional file 4. In general, there was a moderately high correspondence between both nosologies for the most and least severe inhalant use disorder classifications (i.e., DSM-IV inhalant Dependence-LCA highly symptomatic use and DSM-IV no inhalant diagnosis-LCA nonsymptomatic). It is also notable that DSM-IV tended to classify more inhalant users into both the inhalant dependence (28.3%) and no inhalant abuse or dependence disorder (53.0%) groups, than the LCA-derived nosology assigned to their highly symptomatic (24.7%) and nonsymptomatic (47.0%) group counterparts. Significantly less correspondence was observed for the DSM-IV inhalant abuse-LCA symptomatic group. The LCA-based nosology was significantly more likely to assign an inhalant user to the symptomatic group than the DSM-IV nosology was to assign an inhalant abuse diagnosis (28.3% vs. 18.6%).

Of the 52 inhalant users meeting DSM-IV NOS criteria (i.e., who did not meet full diagnostic criteria for either inhalant abuse or dependence, but who met 1 or 2 inhalant dependence criteria), 14 (26.9%) were assigned to the LCA symptomatic group and 38 (73.1%) to the LCA-nonsymptomatic group. Thus, discrepancies between the DSM-IV and LCA-derived nosologies are attributable, in part, to the greater sensitivity of the LCA-based nosology to detect of youth with significant inhalant-related problems that were subthreshold for receipt of DSM-IV inhalant abuse or dependence diagnoses.

The validity of DSM-IV inhalant abuse and dependence disorders was assessed using LCA. This procedure involved subjecting all inhalant users' responses (yes/no) to DSM-IV inhalant abuse and dependence criteria to LCA including only youth who were diagnosed with DSM-IV inhalant abuse or dependence. The model was restricted, *a priori*, to a two-class solution to determine whether a clear distinction between disorders of abuse and dependence could be discerned. The overall model exhibited a good fit to the data (BIC = 1575, Entropy = .77).

**Figure 2:**
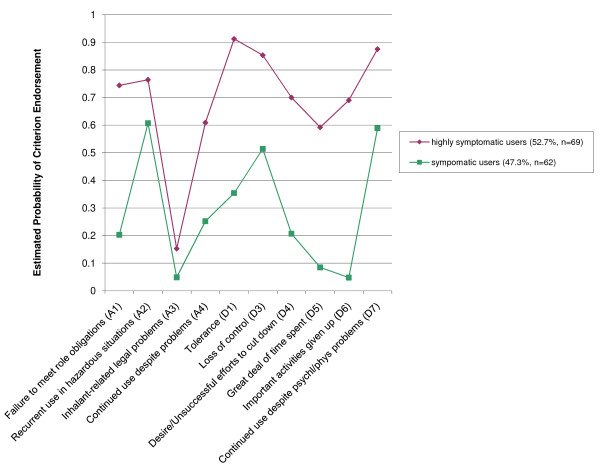
**Latent Class Analysis (Constrained to 2-Group Solution) of Adolescent Inhalant Users' (N = 131) Responses to DSM-IV Inhalant Use Disorder Diagnostic Criteria (note: all inhalant users in this analysis met criteria for either DSM-IV inhalant abuse or dependence)**.

The estimated probabilities of youth in these two classes endorsing each diagnostic criterion are presented in Figure 2. The two groups derived via LCA exhibited distinct ordinally-differentiated sets of estimated probabilities of endorsing DSM-IV IUD criteria. Approximately 52.7% of inhalant users were assigned to the class with higher estimated probabilities of criterion endorsement across all IUD diagnostic criteria, which was again labeled "highly symptomatic inhalant users." Approximately 47.3% of inhalant users with IUDs were assigned to the class with lower estimated probabilities of IUD diagnostic criterion endorsement and were labeled "symptomatic users." Inhalant users were assigned to their respective classes based on the LCA model and then the LCA and DSM-IV-based classifications were compared across inhalant users. Seventy-seven subjects were assigned to the highly symptomatic class using LCA; 59 (74.7%) of these youth met DSM-IV criteria for inhalant dependence disorder, the remainder met DSM-IV inhalant abuse criteria. Sixty-four subjects were assigned to symptomatic inhalant user group via LCA, 42 (80.8%) of these youth met DSM-IV criteria for inhalant abuse; the remaining 19.2% were diagnosed with DSM-IV-defined inhalant dependence.

## Discussion

### Summary of Findings

Inhalant use disorders were found at a considerably higher level in this large sample of juvenile justice system-involved youth than generally found in community samples of adolescents. Of the 279 (38.5%) youth who reported lifetime inhalant use, 131 (47.0%) met criteria for a DSM-IV IUD. Thus, the overall burden of DSM-IV IUDs in this population is estimated at 18.1%, with nearly one-in-five youth affected by these disorders.

That nearly one-half of all adolescent inhalant users developed formal DSM-IV IUDs further suggests that the abuse liability of inhalants among youth in the juvenile justice system is much higher than would be expected on the basis of prior studies of community and clinical populations of inhalant users [29,30]. Historically, inhalants have been thought to have low abuse liability, although this assumption has to some extent been based on findings from large national surveys of inhalant use that excluded institutional populations of delinquent and psychiatrically-disordered youth. Although the findings of this investigation suggest that IUDs are prevalent in this juvenile justice client population, it is also possible that Missouri youth more generally are at high risk for IUDs. Ridenour, Bray, and Cottler [34] recently reported that 40.3% of the 162 adolescent/young adult (M = 20.3, SD = 2.4) community-based inhalant users they studied in St. Louis, Missouri met criteria for an IUD.

In this study of adolescent inhalant users, inhalant dependence was more prevalent than abuse (Dependence: 28.3% vs. Abuse: 18.6%), whereas Ridenour et al [34]. found that 12.4% of the adolescent/young adult inhalant users they studied met inhalant dependence criteria and 27.9% met inhalant abuse criteria. Further, in this study four-of-five youth with lifetime inhalant abuse did not meet criteria for past-year inhalant abuse. Thus, inhalant abusers appeared to have largely discontinued problematic inhalant use. Conversely, most youth who were diagnosed with lifetime inhalant dependence also met past-year criteria for inhalant dependence, suggesting that inhalant dependence was persistent in its course following onset or had developed within the year prior to assessment. In the absence of data pertaining to age at onset of inhalant dependence, it was not possible to determine the proportions of inhalant dependent youth who were persistently inhalant dependent over several years versus the proportion with recent (i.e., past year) onset of inhalant dependence who may or may not become persistently inhalant dependent. Nonetheless, these findings suggest that inhalant dependent youth may be a discrete group that could benefit from targeted interventions of an intensive nature.

Given the young average age of the sample, the finding that nearly 90% of youth with lifetime inhalant dependence met past-year criteria for inhalant dependence, and that many dependent youth also met DSM-IV inhalant abuse criteria, it is possible that a significant number of adolescent inhalant users transitioned from inhalant abuse to dependence in their middle-teenage years. Future studies should examine the timing and patterns of transitions between inhalant use, abuse, and dependence.

With the exception of legal problems, specific signs and symptoms of IUDs were prevalent in this adolescent population. For example, 23.7%, 32.6%, 5.0%, and 21.1% of inhalant users met role failure, hazardous use, legal problems, and social problems inhalant abuse criteria. Comparable figures for Ridenour et al.'s [34] sample of community-based adolescent/young adult inhalant users were: 8.9%, 24.8%, 0.0%, and 8.9%. Thus, the general pattern of findings for inhalant abuse criteria was similar for inhalant users in the current study and Ridenour et al.'s study of community-based inhalant users, but prevalence rates were notably higher among youth in this juvenile justice sample.

In this study and Ridenour et al.'s [34] investigation, continued inhalant use despite inhalant-related physical and psychological problems and inhalants taken in larger amounts or longer than intended, were among the most prevalent inhalant dependence criteria among inhalant users (albeit rates were higher in this sample). However, other dependence symptoms were much more prevalent among inhalant users in this investigation compared to the study conducted by Ridenour et al [34]. These findings suggest that problematic inhalant use and formal IUDs are comparatively prevalent among delinquent youth.

### Comparison of LCA and DSM-IV Classifications

#### Diagnostic concordance

Latent class analyses of DSM-IV inhalant abuse and dependence criteria yielded clearly identifiable, ordinally-differentiated patterns of criterion endorsement for nonsymptomatic, symptomatic and highly symptomatic inhalant users [34,35]. Further, a moderately-high level of correspondence between the LCA-derived and DSM-IV-assigned classifications was observed, particularly for the LCA highly symptomatic-DSM-IV dependence and LCA nonsymptomatic-DSM-IV no diagnosis groups. DSM-IV inhalant abuse diagnoses were much less concordant with LCA-based diagnoses than other DSM-IV inhalant-related diagnoses.

DSM-IV inhalant abuse and dependence diagnoses appeared to distinguish two groups of persons with inhalant-related problems differing primarily in severity, although the specific abuse and dependence criteria may function differently than conceptualized in DSM-IV. Specifically, DSM-IV inhalant abuse and dependence diagnoses did not appear to divide problem inhalant users into discrete groups with high abuse-low dependence and low abuse-high dependence symptom profiles. Rather, across all DSM-IV IUD criteria – abuse and dependence – LCA-defined highly symptomatic users had higher rates of symptom endorsement than did LCA-symptomatic users. To what extent these findings are attributable to the hierarchical nature of the DSM-IV inhalant abuse and dependence diagnoses, which do not allow persons to receive an inhalant abuse diagnosis if they have received an inhalant dependence diagnosis, but do allow persons diagnosed with inhalant dependence to also meet an inhalant abuse diagnosis is not clear. The fact that three criteria must be met to receive an inhalant dependence diagnosis (compared to one for inhalant abuse) and the hierarchical nature of the inhalant dependence diagnosis, undoubtedly contribute to the notable differences between inhalant users meeting abuse and dependence criteria with regard to problem severity.

#### Criterion-level analyses

At the diagnostic level, results summarized above suggest potential problems with the conceptualization and/or assessment of DSM-IV inhalant abuse. Criterion-level findings provided further indication that some inhalant abuse criteria may lack discriminant validity. Study findings suggested that DSM-IV inhalant dependence criteria performed well in distinguishing nonsymptomatic, symptomatic, and highly symptomatic inhalant users in both LCA analyses. However, when examining all inhalant users, inhalant abuse criteria did not perform well in differentiating nonsymptomatic and symptomatic inhalant users. Additionally, when examining only subjects who met DSM-IV criteria for abuse or dependence, two of four inhalant abuse criteria (i.e., recurrent use in hazardous situations and inhalant-related legal problems) did not clearly distinguish symptomatic and highly symptomatic inhalant users. These findings are consistent with those of other studies that suggest abuse symptoms, such as recurrent use in hazardous situations and substance-related legal problems, do not effectively discriminate problem severity [25].

#### Reasons for Discrepancies

It is important to consider factors that might account for discrepancies between diagnostic approaches. One explanation relates to the significant heterogeneity among inhalant users in general and within the DSM-IV constructed groups specifically. Like other studies of inhalant use, this study did not account for types or modes of inhalant use, which can lead to fundamentally different biological, social, and psychological consequences. Prior descriptive studies with this sample of delinquent youth revealed a wide range of inhalants used and modes of use, in addition to high rates of polyinhalant use [27]. Thus, it is reasonable to expect varied clinical profiles in the sample. Because the DSM-IV classifies individuals using a polythetic formulation (i.e., a proportion of the overall set of diagnostic criteria must be met to receive a given diagnosis), whereas the LCA seeks individuals with similar patterns in response sets, some discrepancies are expected.

Another potential explanation for discrepancies between the two diagnostic approaches relates to the conceptualization of problematic inhalant use. The DSM-IV classifies abuse and dependence as separate disorders. This classification was supported, in part, with the LCA. However, the underlying assumption of the DSM-IV classification is that the proper way to model problematic inhalant use is assuming a categorical variable when, in fact, the problem may be continuously distributed. Thus, both DSM-IV and LCA are prone to error if they are classifying people with a disorder that exists on a theoretical continuum. This is not to argue against the utility of a categorical formulation, but to highlight the sources that may account for discrepancies between the two approaches. Future research should examine problematic inhalant use as a continuous variable using both factor analytic methods and item response theory to better understand the nature of IUDs and how they should be measured.

Discrepancies between diagnostic approaches might also be due to the relatively large group of diagnostic orphans, who evidenced one or two signs/symptoms of inhalant dependence but who were not classified as having an IUD when DSM-IV criteria were applied. The existence of a sizable group of diagnostic orphans with clinically significant inhalant dependence signs and symptoms that go undiagnosed under current DSM-IV diagnostic protocols could be one argument in favor of a unidimensional approach to the conceptualization and assessment of inhalant use disorders (i.e., dependence).

### Limitations and Future Directions

Limitations of this report include the self-report nature of the data (biochemical assays are currently largely unavailable or infeasible for inhalant use assessment), and uncertain reliability of the DIS assessment of DSM-IV IUD diagnostic criteria. Few studies of the reliability of DSM-IV IUD assessments have been reported [34,36]. The DIS is one of the oldest and most well established measures of DSM-IV substance use disorders, but caution is urged in the interpretation of these findings until additional reliability studies of DIS-assessed DSM-IV IUDs are performed. Another key limitation of the reported research is that the authors' cannot place IUDs in the context of other DSM-IV substance use disorders or non-substance-related psychiatric disorders because these conditions were not assessed via structured psychiatric interview in this inhalant-focused study. Information about comorbid substance use and psychiatric disorders could help guide treatment responses and enhance the efficacy of inhalant treatment interventions. Finally, although the antisocial nature of the sample we examined might limit the generalizability of reported findings, we note that antisocial youth are an important and understudied population in their own right, antisocial behavior is a common concomitant of substance use disorders in the general population, and that our sample included the full gamut of antisocial youth, ranging from those with minor behavioral problems to the severely antisocial.

## Conclusion

IUDs and several individual IUD criteria were prevalent in this sample of antisocial youth. A number of youth met one or two inhalant dependence criteria but did not meet DSM-IV criteria for inhalant abuse or dependence. These diagnostic orphans evidenced worrisome inhalant-related problems that are not captured by the current set of IUD diagnostic criteria in DSM-IV. Study findings provided evidence for distinct classes of symptomatic and highly symptomatic inhalant users identified via latent class analyses of DSM-IV inhalant abuse and dependence criteria. Relatively high levels of correspondence were identified for LCA-derived and DSM-IV-defined diagnoses of LCA-highly symptomatic inhalant use/DSM-IV inhalant dependence and LCA nonsymptomatic/DSM-IV no diagnosis groups. Data were less supportive at the diagnostic and criterion levels of the validity of the DSM-IV abuse diagnosis. The ability to identify groups of inhalant users differing in the severity of their IUDs may be important for treatment purposes. The results of this investigation establish the need for IUD screening, prevention, and treatment interventions directed to the more than one million youth who are annually involved with the juvenile justice system. Future research should also focus on the question of how wide the nomological net should be cast in an effort to capture all youth displaying inhalant-related problems in contemporary diagnostic schemes.

## Competing interests

Neither author has a financial interest or relationship with an individual or organizational entity that constitutes a conflict of interest with regard to the subject of this manuscript.

## Authors' contributions

MOH drafted the manuscript and formulated analyses. BEP formulated and conducted statistical analyses, edited the manuscript, and also helped draft the manuscript. Both authors take responsibility for the integrity of the data and the accuracy of the data analysis and both authors had full access to the data in the study. Both authors read and approved the final manuscript.

## Pre-publication history

The pre-publication history for this paper can be accessed here:



## Supplementary Material

Additional file 1 **Number and Proportion of Delinquent Adolescents Meeting Each Lifetime DSM-IV Inhalant Abuse Criterion in the Overall Sample, Subsample of Lifetime Inhalant Users, and Subsamples of Youth Meeting DSM-IV Lifetime Inhalant Abuse and Inhalant Dependence Criteria.** Click here for file

Additional file 2 **Number and Proportion of Delinquents Meeting Each DSM-IV Inhalant Dependence Criterion in the Overall Sample, Subsample of Lifetime Inhalant Users, and Subsamples of Youth Meeting DSM-IV Inhalant Dependence and Inhalant Abuse Diagnostic Criteria or Inhalant Use Disorder Not Otherwise Specified.** Click here for file

Additional file 3 **Empirical Fit of 1-, 2-, 3- and 4-Class Models and Class Sizes Based on Latent Class Analysis of DSM-IV Inhalant Use Disorder Diagnostic Criteria.** Click here for file

Additional file 4 **Correspondence between DSM-IV-defined and LCA-derived inhalant use disorder diagnoses in delinquent inhalant users.** Click here for file
