# Supplementary material for: A Survey of Inhalant Use Disorders among Delinquent Youth: Prevalence, Clinical Features, and Latent Structure of DSM-IV Diagnostic Criteria
Source: BMC Psychiatry. 2009 Mar 8;9:8. doi: 10.1186/1471-244X-9-8 (PMC2657136; doi:10.1186/1471-244X-9-8)
Supplement: Additional file 1 — Number and Proportion of Delinquent Adolescents Meeting Each Lifetime DSM-IV Inhalant Abuse Criterion in the Overall Sample, Subsample of Lifetime Inhalant Users, and Subsamples of Youth Meeting DSM-IV Lifetime Inhalant Abuse and Inhalant Dependence Criteria. [file 1471-244X-9-8-S1.doc]

Table 1. Number and Proportion of Delinquent Adolescents Meeting Each Lifetime DSM-IV Inhalant Abuse Criterion in the Overall Sample, Subsample of Lifetime Inhalant Users, and Subsamples of Youth Meeting DSM-IV Lifetime Inhalant Abuse and Inhalant Dependence Criteria.

| DSM-IV Inhalant Abuse  Diagnostic Criteria | Overall sample*  N = 723  N (%) | Lifetime Inhalant Users  N = 279  N (%) | Lifetime Inhalant Abuse  N = 52  N (%) | Lifetime Inhalant Dependence  N = 79  N (%) |
| --- | --- | --- | --- | --- |
| 1. Recurrent inhalant use resulting in a failure to fulfill role obligations at home, work, or school | 66 (9.1) | 66 (23.7) | 17 (32.7) | 49 (62.0) |
| 1. Recurrent inhalant use in situations in which is it physically hazardous | 91 (12.6) | 91 (32.6) | 37 (71.1) | 54 (68.4) |
| 1. Recurrent inhalant-related legal problems | 14 (1.9) | 14 (5.0) | 4 (7.7) | 10 (12.7) |
| 1. Continued inhalant use despite having persistent or recurrent social or interpersonal problems caused or exacerbated by the effects of the inhalant(s) | 59 (8.2) | 59 (21.1) | 15 (28.9) | 44 (55.7) |

*Except for the abuse and dependence columns, categories identified in table columns are not mutually exclusive.”
