# Supplementary material for: A Survey of Inhalant Use Disorders among Delinquent Youth: Prevalence, Clinical Features, and Latent Structure of DSM-IV Diagnostic Criteria
Source: BMC Psychiatry. 2009 Mar 8;9:8. doi: 10.1186/1471-244X-9-8 (PMC2657136; doi:10.1186/1471-244X-9-8)
Supplement: Additional file 2 — Number and Proportion of Delinquents Meeting Each DSM-IV Inhalant Dependence Criterion in the Overall Sample, Subsample of Lifetime Inhalant Users, and Subsamples of Youth Meeting DSM-IV Inhalant Dependence and Inhalant Abuse Diagnostic Criteria or Inhalant Use Disorder Not Otherwise Specified. [file 1471-244X-9-8-S2.doc]

Table 2. Number and Proportion of Delinquents Meeting Each DSM-IV Inhalant Dependence Criterion in the Overall Sample, Subsample of Lifetime Inhalant Users, and Subsamples of Youth Meeting DSM-IV Inhalant Dependence and Inhalant Abuse Diagnostic Criteria or Inhalant Use Disorder Not Otherwise Specified.

| DSM-Inhalant Dependence  Diagnostic Criteria | Overall sample**  N = 723  N (%) | Lifetime  Inhalant Users  N = 279  N (%) | Lifetime Inhalant Abuse‡  N=52  N (%) | Lifetime Inhalant Dependence^†^  N = 79  N (%) | Lifetime Inhalant Use Disorder NOS^§^  N= 52  N (%) |
| --- | --- | --- | --- | --- | --- |
| 1. Tolerance* | 127 (17.6) | 127 (45.5) | 17 (32.7) | 70 (88.6) | 17 (32.7) |
| a. A need for markedly increased amounts of the inhalant(s) to achieve intoxication or desired effect | 111 (15.4) | 111 (39.8) | 15 (28.9) | 65 (82.3) | 12 (23.1) |
| b. Markedly diminished effect with continued use of the same amount of the inhalant(s) | 92 (12.7) | 92 (33.0) | 11 (21.2) | 54 (68.4) | 12 (23.1) |
| 3. The inhalant(s) is often taken in larger amounts or for a longer period of time than was intended | 127 (17.6) | 127 (45.5) | 23 (44.2) | 69 (87.3) | 13 (25.0) |
| 4. There is a persistent desire or unsuccessful efforts to cut down or control inhalant use | 102 (14.1) | 102 (36.6) | 14 (26.9) | 49 (62.0) | 19 (36.5) |
| 5. A great deal of time is spent in activities necessary to obtain the inhalant, use the inhalant, or recover from its effects | 60 (8.3) | 60 (21.5) | 5 (9.6) | 43 (54.4) | 4 (7.7) |
| 6. Important social, occupational, or recreational activities are given up or reduced because of inhalant use. | 60 (8.3) | 60 (21.5) | 7 (13.4) | 46 (58.2) | 1 (1.9) |
| 7. Inhalant use is continued despite knowledge of having a persistent or recurrent physical or psychological problem that is likely to have been caused or exacerbated by the inhalant(s) | 124 (17.5) | 124 (44.4) | 27 (51.9) | 71 (89.9) | 14 (26.9) |

Note: *Values for the Tolerance criterion are based on the number of respondents who reported either sub-criterion A or sub-criterion B or both. **Except for the abuse, dependence, and inhalant use disorder NOS columns, categories identified in table columns are not mutually exclusive.” ^†^Inhalant Dependence diagnosis reflects the presence of 3 or more Inhalant Dependence signs/symptoms clustering in one 12-month period over a youth’s lifetime. ‡Diagnosis reflects the presence of 1 or more of 4 DSM-IV Inhalant Abuse signs/symptoms clustering in one 12-month period over a youth’s lifetime. Youth who met inhalant dependence criteria were not eligible for an inhalant abuse diagnosis. ^§^ = Inhalant Use Disorder Not Otherwise Specified (NOS) which was defined as meeting either 1 or 2 lifetime inhalant dependence symptoms but meeting diagnostic criteria for neither Inhalant Abuse or Inhalant Dependence Disorders. Persons evidencing this pattern of responses are often referring to as “diagnostic orphans.”
