# Supplementary material for: A Survey of Inhalant Use Disorders among Delinquent Youth: Prevalence, Clinical Features, and Latent Structure of DSM-IV Diagnostic Criteria
Source: BMC Psychiatry. 2009 Mar 8;9:8. doi: 10.1186/1471-244X-9-8 (PMC2657136; doi:10.1186/1471-244X-9-8)
Supplement: Additional file 3 — Empirical Fit of 1-, 2-, 3- and 4-Class Models and Class Sizes Based on Latent Class Analysis of DSM-IV Inhalant Use Disorder Diagnostic Criteria. [file 1471-244X-9-8-S3.doc]

Table 3. Empirical Fit of 1-, 2-, 3- and 4-Class Models and Class Sizes Based on Latent Class

Analysis of DSM-IV Inhalant Use Disorder Diagnostic Criteria

| Class Solution | BIC | Entropy | Class, n (%) |
| --- | --- | --- | --- |
| 1 Class | 3212 | NA | 279 (100%) |
| 2 Classes | 2713 | .836 | 104.5 (37.4)  174.5 (62.6) |
| 3 Classes | 2679 | .825 | 70.2 (25.2)  78.4 (28.1)  130.3 (46.7) |
| 4 Classes | 2690 | .859 | 60.4 (21.6)  37.1 (13.3)  50.4 (18.1)  131.2 (47.0) |
