# Supplementary material for: A Survey of Inhalant Use Disorders among Delinquent Youth: Prevalence, Clinical Features, and Latent Structure of DSM-IV Diagnostic Criteria
Source: BMC Psychiatry. 2009 Mar 8;9:8. doi: 10.1186/1471-244X-9-8 (PMC2657136; doi:10.1186/1471-244X-9-8)
Supplement: Additional file 4 — Correspondence between DSM-IV-defined and LCA-derived inhalant use disorder diagnoses in delinquent inhalant users. [file 1471-244X-9-8-S4.doc]

Table 4. Correspondence between DSM-IV-defined and LCA-derived inhalant use disorder diagnoses in delinquent inhalant users

|  | LCA-Highly Symptomatic | LCA Symptomatic | LCA Non-symptomatic | Total |
| --- | --- | --- | --- | --- |
| DSM-IV No Diagnosis  n  (row %)  (column %) | 0  (0)  (0) | 26  (27.1)  (32.9) | 70  (72.9)  (53.4) | 96  -  (34.4) |
| DSM-IV NOS  n  (row %)  (column %) | 0  (0)  (0) | 14  (26.9)  (17.7) | 38  (73.1)  (29.0) | 52  -  (18.6) |
| DSM-IV Lifetime Abuse  n  (row %)  (column %) | 15  (28.8)  (21.7) | 14  (26.9)  (17.7) | 23  (44.2)  (17.6) | 52  -  (18.6) |
| DSM-IV Lifetime Dependence  n  (row %)  (column %) | 54  (68.3)  (78.3) | 25  (31.6)  (31.6) | 0  (0)  (0) | 79  -  (28.3) |
| Total  n  (row%)  (column%) | 69  (24.7)  - | 79  (28.3)  - | 131  (47.0)  - | 279  (100)  (100) |
